# Journal of translational medicine reviewer acknowledgement 2012

**DOI:** 10.1186/1479-5876-11-55

**Published:** 2013-03-20

**Authors:** Francesco Marincola

**Affiliations:** 1 Sidra Medical and Research Center, Doha, Qatar

## Abstract

**Contributing reviewers:**

The editor of *Journal of Translational Medicine* would like to thank all our reviewers who have contributed to the journal in Volume 10 (2012).

## 

Michał Żmijewski

Poland

Sonia Abdelhak

Tunisia

Haitham Abou Saleh

Qatar

Raffaele Addeo

Italy

Gail Adler

United States of America

Xabier Agirre

Spain

Aamir Ahmad

United States of America

Bassil Akra

Germany

Erdem Aktas

Turkey

Safiye Aktas

Turkey

Issa Al Salmi

Oman

Ayodele Alaiya

Saudi Arabia

Balbino Alarcon

Spain

Abla Albsoul-Younes

Jordan

Mariam AL-Muftah

Qatar

Fahd Al-Mulla

Kuwait

Cestmir Altaner

Slovakia

Cristina Alvira

United States of America

Yihua An

China

Daniel Anderson

United States of America

Roland Andersson

Sweden

Sébastien Anguille

Belgium

Javier Angulo

Spain

Eleni Anni

United States of America

Stephane Ansieau

France

Jedrzej Antosiewicz

Poland

Ichiro Aoki

Japan

Jaime Arias

Spain

M. Simo Arredouani

United States of America

Paolo A. Ascierto

Italy

Maria Libera Ascierto

United States of America

Esther Aspinall

United Kingdom

Stephen Atkin

United Kingdom

Michaela Aubele

Germany

Walter Aulitzky

Germany

Dorina Avram

United States of America

Alexander Awgulewitsch

United States of America

Caner Aytekin

Turkey

Luciano Cesar Azevedo

Brazil

N. Ahmad Aziz

Netherlands

Hideo Baba

Japan

Juan Badimon

United States of America

Abeer Bahnassy

Egypt

Xiaowen Bai

United States of America

Sasha Bakhru

United States of America

Alberto Balbarini

Italy

Marcelo Baldo

United States of America

Marija Balic

Austria

Arun Bandyopadhyay

India

Probal Banerjee

United States of America

Mohamed Banni

Tunisia

Rathindranath Baral

India

David Barnett

United Kingdom

Jonathan Barratt

United Kingdom

Guislaine Barriere

France

Esther Bastiaannet

Netherlands

Marcus Bauer

Germany

Andrew Bauer

United States of America

Constantin Baxevanis

Greece

Therese Becker

Australia

Christian Becker

Germany

Natalia Bednarz-Knoll

Germany

Davide Bedognetti

United States of America

Shahinaz Bedri

Qatar

Cristobal Belda-Iniesta

Spain

Matteo Bellone

Italy

Andreas Bembenek

Germany

Ahlem Ben Haj Ayed

Tunisia

Rotem Ben-Hamo

Israel

Marc Benhamou

France

Tawfeg Ben-Omran

Qatar

Michèle Bergeron

Canada

Myriam Bernaudin

France

Laia Bernet Vegue

Spain

Harold Bernstein

United States of America

Emanuele Bertaglia

Kazakhstan

Cyril Berthet

France

Brian Bettencourt

United States of America

Karen Bieback

Germany

Francesco Bifari

Italy

Mark Bigham

Canada

Ralf Birkenhäger

Germany

Patrick Biston

Belgium

Maria Bjorkqvist

Sweden

Beata Bode

Switzerland

Stefan Boehm

Austria

Fausto Bogazzi

Italy

Paul Bogner

United States of America

Johannes Boltze

Germany

Bruno Bonetti

Italy

Francesca Bonvicini

Italy

Cesar Borlongan

United States of America

Francesc E. Borràs

Spain

Paolo Boscolo-Rizzo

Italy

Demosthenes Bouros

Greece

Jason Bovenkerk

United States of America

Onur Boyman

Switzerland

Peter Brader

Austria

Valdir Braga

Brazil

Karim Brandt

Switzerland

Andy Brass

United Kingdom

Hiltrud Brauch

Germany

Michel Braun

Belgium

Marco Bregni

Italy

Ekua Brenu

Australia

Emilio Bria

Italy

Kristie Bridges

United States of America

Michael Briggs

United States of America

Thomas Brinker

United States of America

Cedrik M. Britten

Germany

Tom Broderick

United States of America

Vincenzo Bronte

Italy

David Brown

United Kingdom

Robert Brown

United Kingdom

Lindsay Brown

Australia

Richard Bruce

United States of America

Boglarka Brugos

Hungary

Hermann Brustmann

Austria

Donald Buchsbaum

United States of America

Alfredo Budillon

Israel

Kimberly Bullock

United States of America

Franco Maria Buonaguro

Italy

Luigi Buonaguro

Italy

Stefan Burdach

Germany

Dennis Burton

United States of America

James Bussel

United States of America

Pierre Busson

France

Marcus Butler

Canada

Lisa Butterfield

United States of America

Neslihan Cabioglu

Turkey

Danielle Camisasca

Brazil

Irene Cantarero

Spain

Michele Caraglia

Italy

Michela Carlet

Germany

Peter Carlsson

Sweden

Antoine Carpentier

France

Robert Carr

United States of America

William Carson

United States of America

Lorraine Caruccio

United States of America

Luciano Castiello

United States of America

Jorge Castillo

United States of America

Diane Cathy

United Kingdom

Antonino Cattaneo

Italy

Charles Cazanave

France

Jonathan Cebon

Australia

Laura Cerezo

Spain

Runu Chakravarty

India

Marc Chamberlain

United States of America

Jun Chang Duk

Korea, South

Boonsri Chanrachakul

Thailand

Aude Chapuis

United States of America

Prabal Chatterjee

United Kingdom

Yuh-Ling Chen

Taiwan

Zhe-Sheng Chen

United States of America

Wen-Shiang Chen

Taiwan

Ya-Wen Chen

Taiwan

Miaofen Chen

Taiwan

Kuen-Feng Chen

Taiwan

George Chen

Hong Kong

Wenqiang Chen

China

Liang Cheng

United States of America

SK Cheong

Malaysia

Jason Chesney

United States of America

Rémi Cheynier

France

Harvinder Singh Chhabra

India

Maurizio Chiriva-Internati

United States of America

Wee Chng

Singapore

William CS Cho

Hong Kong

Thinle Chodon

United States of America

Murim Choi

United States of America

Benjamin Chong

United States of America

Jeffrey Chou

United States of America

Lotfi Chouchane

Qatar

Sara Civini

United States of America

Giovanni Cizza

United States of America

Jessica Clement

United States of America

Martine Clozel

Switzerland

Everardo Cobos

United States of America

Dawn Cochrane

United States of America

Samuel M. Cohen

United States of America

William Coleman

United States of America

Robert Coleman

United Kingdom

Pablo Conesa-Zamora

Spain

Richard Connolly

United States of America

Elena Conti

Italy

Jonathan Cools-Lartigue

Canada

Pierre Cordelier

France

Pierpaolo Correale

Italy

Winston Costa Pereira

India

Alain Couvineau

France

Tommy J Curry

United States of America

Ales Cvekl

United States of America

Gregory Czarnota

Canada

Maria Dagli

Brazil

Yang Dai

United States of America

Zhong-Min Dai

China

Wangde Dai

United States of America

Ashraf Dallol

Saudi Arabia

Felipe Dal-Pizzol

Brazil

Ravi Dasu

United States of America

Nabanita Datta

United States of America

Adil Daud

United States of America

Simon Davis

United Kingdom

Jonathan D'Cunha

United States of America

Andreia De Albuquerque

Germany

Rob De Boer

Netherlands

Ugo De Giorgi

Italy

Renske De Jong

Netherlands

Andrea De Maria

Italy

Katleen De Preter

Belgium

Valli De Re

Italy

Ilaria Decimo

Italy

Mario Delgado

Spain

A. Lee Dellon

United States of America

Sandra Demaria

United States of America

Aldona Dembinska-Kiec

Poland

Yubin Deng

China

Jing-Yu Deng

China

Rajat Deo

United States of America

Sebastien Deshayes

France

Joseph Desimone

United States of America

Susan Deutscher

United States of America

Animesh Dhar

United States of America

Maria Di Bartolomeo

Italy

Giuseppe Di Lorenzo

Italy

Stefano Di Santo

Switzerland

Yinze Diao

China

Valdo Jose Dias Da Silva

Brazil

Giorgio Dieci

Italy

Susanne Dihlmann

Germany

Ioanna Dimopoulou

Greece

Hong Ding

Qatar

Mary Disis

United States of America

Soner Dogan

United States of America

Elena Dogliotti

Italy

Alexander Dohnal

Austria

Alena Donda

Switzerland

Glenn Dorsam

United States of America

Xiang Du

China

Erin Dunbar

United States of America

Jeffery Dusek

United States of America

Robert Edwards

United States of America

Thomas Efferth

Germany

Nejat Egilmez

United States of America

Brian Eisner

United States of America

Miquel Ekkelenkamp

Netherlands

Heather Eliassen

United States of America

Petrescu Emil

Romania

Felix Engel

Germany

Masaru Enomoto

Japan

Maria Eriksdotter

Sweden

Kristina Eriksson

Sweden

Jose Esparza

United States of America

Markus Essler

Germany

James Faix

United States of America

Mario Falchi

United Kingdom

Buwu Fang

China

Bingliang Fang

United States of America

Haneen Farah

Israel

Karim Farhat

Saudi Arabia

Mark Faries

United States of America

Ramon Farre

Spain

Peter Fasching

Germany

Hassana Fathallah

United States of America

Maria Simonetta Faussone-Pellegrini

Italy

Jou Felsberg

Germany

Jane Ferguson

United States of America

Daris Ferrari

Italy

Silvano Ferrini

Italy

Soldano Ferrone

United States of America

Nikos Fersis

Germany

Laszlo Fesus

Hungary

Georg Martin Fiedler

Switzerland

Jonine Figueroa

United States of America

Pier Luigi Fiorella

Italy

Massimo Fiorilli

Italy

Paul Fisher

United States of America

Ffolliott Fisher

United Kingdom

Tom Flanagan

Ireland

Jane Flint

United States of America

Carlos Flores

Spain

Franco Folli

United States of America

Dominic Fong

Italy

Bernard Fox

United States of America

Cesari Francesca

Italy

Rodrigo Franco

United States of America

Richard Frank

United States of America

Kai Fu

United States of America

Etsuko Fujii

Japan

Minoru Fukuchi

Japan

Tsuyoshi Fukushima

Japan

Kengo Furuichi

Japan

Tatsuhiko Furukawa

Japan

Michael Gabriel

Austria

Thomas Gajewski

United States of America

Sanjeev Galande

India

Maria Galindo

Spain

Maurizio Gallo

Italy

Giovanni Gambaro

Italy

Dashiell Gantner

Australia

Giacomo Garibotto

Italy

Stephen Gately

United States of America

Sebastiano Gattoni-Celli

United States of America

Subhash Gautam

United States of America

Carsten Geisler

Denmark

Cicek Gercel-Taylor

United States of America

Thomas Gevaert

Belgium

Alexander Ghanem

Germany

Shobha Ghosh

United States of America

Luca Giacomelli

Italy

Gianluigi Giannelli

Italy

Geoffrey Gibney

United States of America

M Ian Gilmour

United States of America

Elisa Giovannetti

Italy

Paul Gissen

United Kingdom

Gabriella Gobbi

Canada

Sayed Goda

United Kingdom

Alessandra Godoy

Brazil

Jacky Goetz

France

David Goldfarb

United States of America

Eny Maria Goloni Bertollo

Brazil

Carmen Gonzalez

Spain

Walter Gotlieb

Canada

Stephen Gottschalk

United States of America

Michael Grandner

United States of America

William B. Grant

United States of America

Catherine Gratas-Rabbia-Ré

France

Iris Groeneveld

Netherlands

David Gross

Israel

Kenneth Grossmann

United States of America

Jin Gu

China

Jun Gu

China

Luigina Guasti

Italy

Gerardo Guiter

Qatar

Maya Gulubova

Bulgaria

Jolanta Gutkowska

Canada

Balazs Györffy

Hungary

Mandana Haack-Sørensen

Denmark

John Haanen

Netherlands

Michael Haase

Germany

Tillie-Louise Hackett

Canada

Joerg Haier

Germany

Niels Halama

Germany

Hanan Hamamy

Switzerland

Michal Hammel

United States of America

Hind Hamzeh-Cognasse

France

Takahiko Hara

Japan

Alexandre Harari

Switzerland

Pauline Harper

Sweden

Johan Hartman

Sweden

Amanda Harvey

United Kingdom

Elham Hassen

Tunisia

Ilmo Hassinen

Finland

James Hatcher

United States of America

Robert Hawkins

United Kingdom

Douik Hayet

Tunisia

Chao He

China

Ping-An He

China

Grzegorz Helbig

Poland

Richard Heller

United States of America

Peiman Hematti

United States of America

Akseli Hemminki

Finland

Karen Herbst

United States of America

Henry Herce

United States of America

Gonzalo Herradon

Spain

Ken Herrmann

Germany

Helen Heslop

United States of America

Luke Hesson

Australia

Marie-Françoise Heymann

France

Sarah Highlander

United States of America

Harry Hill

United States of America

Hans H Hirsch

Switzerland

Robert Hoffman

United States of America

Yukihiro Hojo

Japan

Bruce Hollis

United States of America

Ulrika Holmlund

Sweden

Andrew Holt

United Kingdom

David Hong

United States of America

Jin Tae Hong

Korea, South

Stephen Hopkins

United Kingdom

Craig Horbinski

United States of America

Raymund E. Horch

Germany

Akira Horii

Japan

Hisanori Horiuchi

Japan

Konstantin-A. Hossmann

Germany

Jian Hou

China

Weihong Hou

United States of America

Peter Houghton

United States of America

Xiaoxia Hu

China

Ke-Qin Hu

United States of America

Yu Huang

China

Fei Huang

United States of America

Dennis Hughes

United States of America

Kam HUI

Singapore

Ianko Iankov

United States of America

Toshihiro Ichiki

Japan

Thomas Ichim

United States of America

Giorgio Iervasi

Italy

Kristiina Iljin

United States of America

Sebastian Illes

Austria

Edward Ingenito

United States of America

Martino Introna

Italy

Naoki Ishiguro

Japan

Jun Iwamoto

Japan

Pawel Jagodzinski

Poland

Smita Jaiswal

United States of America

Anders Jakobsen

Denmark

Rune Jakobsen

Norway

Yeona Jang

Canada

Gabor Jarai

United Kingdom

Tatiana Jazedje

Brazil

Xu Ji

Japan

Junfang Ji

United States of America

Rui-Cheng Ji

Japan

Zhenyu Jia

United States of America

Wei Jia

United States of America

Wang Jian Hua

China

Ping Jin

United States of America

Zi-Bing Jin

Japan

Jianjian Jin

United States of America

Ferenc Jolesz

United States of America

Catherine Jopling

United Kingdom

Jingfang Ju

United States of America

Natsuko Kakudo

Japan

Pawel Kalinski

United States of America

Dimitrios Kalkanis

Greece

Galatea Kallergi

Greece

Eric Kallwitz

United States of America

Michael Kalos

United States of America

Gregory Kaltsas

Greece

Takehiko Kamijo

Japan

Lana Kandalaft

United States of America

Jiuhong Kang

China

Yibin Kang

United States of America

Burcak Karaca

Turkey

Mohammed Kashani-Sabet

United States of America

Sabine Kasimir-Bauer

Germany

Jens Kastrup

Denmark

Masatoshi Kataoka

Japan

Masaru Katoh

Japan

Steven Katz

United States of America

Marc Kaufman

United States of America

Harikumar Kb

India

Marc JNC Keirse

Australia

Stephen Kemp

Canada

Hilary Kenny

Uruguay

Raouf Khalil

United States of America

Anis Khan

United States of America

Amit Khera

United States of America

Sorapop Kiatpongsan

United States of America

Rolf Kiessling

Sweden

Reuben Kim

United States of America

Hyun Ok Kim

Korea, South

Dae-Ghon Kim

Korea, South

Young-Jin Kim

Korea, South

Dae Kim

United Kingdom

Joseph Kim

United States of America

Hyun Ju Kim

Korea, South

Kwangmeyung Kim

Korea, South

Michael King

United States of America

Elmar Kirches

Germany

Lidija Klampfer

United States of America

David Klinke

United States of America

Ann Klopp

United States of America

Konstance Knox

United States of America

Holly Koblish

United States of America

Reinhard Kofler

Austria

Takashi Kohno

Japan

Terumi Kohwi-Shigematsu

United States of America

Seiji Kojima

Japan

Zuzana Kolkova

Sweden

Haruki Komatsu

Japan

Ioanna Koniari

Greece

Loukas Kontovinis

Greece

Igor Koralnik

United States of America

Sawa Kostin

Germany

Vassiliki Kotoula

Greece

Katja Kotsch

Austria

Marek Kovar

Czech Republic

Ivan Koychev

United Kingdom

Tomoaki Kozaki

Japan

Thomas Krausz

United States of America

Tibor Krenacs

Hungary

Nikolaus Kroeger

Germany

Rebekka Krumbach

Germany

Lucia Kucerova

Slovakia

Selim Kuci

Germany

Ralf Kueppers

Germany

Wojtek Kulak

Poland

Ganesh Kumar

United States of America

Ruprecht Kuner

Germany

Ajaikumar Kunnumakkara

United States of America

Taeg Kyu Kwon

Korea, South

Nathalie Labarriere

France

Siegfried Labeit

Germany

Roberto Labianca

Italy

Donato Lacedonia

Italy

Moncef Ladjimi

France

Maode Lai

China

Paula Lam

Singapore

Pavlos Lampropoulos

Greece

Tobias Lange

Germany

Ingrid Langer

Belgium

Zvi Laron

Israel

Bruno Laugel

United Kingdom

Lena Lavie

Israel

Juozas Lazutka

Lithuania

Serge Lebecque

France

Jean Lee

United States of America

Peter Lee

United States of America

Chung Lee

United States of America

Maria Lehtinen

United States of America

Valerio Leoni

Italy

A. Martin Lerner

United States of America

Maciej Lesniak

United States of America

Henri Leuvenink

Netherlands

Bruce Levine

United States of America

Stanley Levinson

United States of America

George Lewis

United States of America

Xiaoqing Li

China

Michelle Li

United States of America

Tao-Sheng Li

Japan

Huabin Li

China

Jinjun Li

China

Yangqiu Li

China

Jun Li

China

Xin Li

China

Zhiyong Liang

China

Wen-Ting Liao

China

Dan Liebermann

United States of America

Michael Liebman

United States of America

Jeong Mook Lim

Korea, South

Liu Limei

China

Shi-Ming Lin

Taiwan

Wen-Chang Lin

Taiwan

Guiting Lin

United States of America

Chih-Pin Liu

United States of America

Nan Liu

China

Rui-Ming Liu

United States of America

Ran Liu

China

Qi Liu

China

Kaiyan Liu

China

Lei Liu

United States of America

Chong Liu

United States of America

Qiao Liu

China

Ingrid Ljuslinder

Sweden

David Loch

Australia

Dan Longo

United States of America

Lucia Lopalco

Italy

Xavier López

Spain

Chris Lowrey

United States of America

Shemin Lu

China

Enrico Lucarelli

Italy

Alessandro Lugli

Switzerland

Lawrence Lum

United States of America

Stefano Luminari

Italy

Troy Lund

United States of America

Wei Luo

China

Claudio Luparello

Italy

Ding Ma

China

Patrick Ma

United States of America

Cristina Maccalli

Italy

Jean-Pierre Mach

Switzerland

Timothy Mackey

United States of America

Mhairi Macrae

United Kingdom

Margaret Madeleine

United States of America

Michael Maes

Thailand

Jared Magnani

United States of America

Wijden Mahfoudh

Tunisia

Michele Maio

Italy

Arindam Maitra

India

Hideyuki J. Majima

Japan

Ajay Maker

United States of America

Konstantinos Makris

Greece

Joel Malek

Qatar

Mirko Manetti

Italy

Ferdinando Mannello

Italy

Paolo Manunta

Italy

Hailei Mao

China

Raphaël Maréchal

Belgium

Claudiu Margaritescu

Romania

Arivusudar Marimuthu

India

Victor Marinescu

United States of America

Svetomir Markovic

United States of America

Reinhard Marks

Germany

Annette Marleau

United States of America

David Marshall

United States of America

Sonya Marshall-Gradisnik

Australia

Tracey Amanda Martin

United Kingdom

Giuseppe Martini

Italy

Enca Martin-Rendon

United Kingdom

Giuseppe Masucci

Sweden

Satohiro Masuda

Japan

Abhishek Mathur

India

Hideo Matsuzaki

Japan

Kate Matthay

United States of America

Niklas Mattsson

Sweden

Miroslava Matuskova

Slovakia

Nicola Maurea

Italy

Nayef Mazloum

Qatar

Todd Mcallister

United States of America

Karen Mccloskey

United Kingdom

J Philip Mccoy

United States of America

Paul Mcdermott

United States of America

David Mckenna

United States of America

Nehal Mehta

United States of America

Muhammed Ashraf Memon

Australia

Philippe Merle

France

Alison Merryweather-Clarke

United Kingdom

Piergiorgio Messa

Italy

Mark Messina

United States of America

Raymond Meyn

United States of America

Rigaud Michel

France

Damijan Miklavcic

Slovenia

Ryan Miller

United States of America

Richard Millis

United States of America

Wei-Ping Min

Canada

Shinya Minatoguchi

Japan

LM Mir

France

Masahiro Mizoguchi

Japan

Simone Mocellin

Italy

Holger Moch Moch

Switzerland

Ali Mojallal

France

David G Molleví

Spain

Vladia Monsurrò

Italy

Augusto Montezano

United Kingdom

Jan-Renier Moonen

Netherlands

Lieve Moons

Belgium

Ronald Moore

Canada

Gil Mor

United States of America

Ernesto Moreno

Cuba

Seiichi Mori

Singapore

Ryuichi Morishita

Japan

Bianca Mostert

Netherlands

Frantisek Mrazek

Czech Republic

Pavlos Msaouel

United States of America

Sumanta Mukherjee

United States of America

Hasan Mukhtar

United States of America

Cindy Munro

United States of America

Yoshiki Murakami

Japan

Antonino Musolino

Italy

Linda Myers

United States of America

Cristina Nadal

Spain

Gabriele Nagel

Germany

Sreejayan Nair

United States of America

Chisato Nakada

Japan

Akihiro Nakagomi

Japan

Tetsuya Nakatsura

Japan

Soha Namazi

Iran

Pier Giorgio Natali

Italy

Urs Nater

Switzerland

Urs Nater

Germany

David Nathanson

United States of America

Clara Natoli

Italy

Daniel Navarini

Brazil

Manuela Nebuloni

Italy

Lucien Nedzi

United States of America

Alessandro Neri

Italy

Paul Ness

United States of America

William Newman

United Kingdom

Julia Newton Bishop

United Kingdom

Susanne Dam Nielsen

Denmark

Teemu Niiranen

Finland

Hans Nijman

Netherlands

Tao Ning

China

Reiki Nishimura

Japan

Shigeru Nishizawa

Japan

Boris Novakovic

Australia

Mikako Obika

Japan

Thomas O'Brien

United States of America

Josiah Ochieng

United States of America

M Sue O'Dorisio

United States of America

Yasushi Oginosawa

Japan

Satoshi Ohtake

United States of America

Richard O'Kennedy

Ireland

Oluwadayo Oluwadara

United States of America

Frank Ondrey

United States of America

Barbaros Oral

Turkey

Antonio Orlacchio

Italy

Augusto Orlandi

Italy

Heidi Ormstad

Norway

Stein Orn

Norway

Marlene Oscar-Berman

United States of America

Atsushi Otani

Japan

Patrick Ott

United States of America

Allal Ouhtit

Oman

Helieh Oz

United States of America

Iwata Ozaki

Japan

Sophie Paczesny

United States of America

Debnath Pal

India

Giuseppe Palmieri

Italy

Giuseppe Palumbo

Italy

Monica C. Panelli

United States of America

Klaus Pantel

Germany

Constantinos Pantos

Greece

Gianpaolo Papaccio

Italy

Luis A Pardo

Germany

Joo-In Park

Korea, South

Jinho Park

Korea, South

Maura Parker

United States of America

Robert Passier

Netherlands

Gregory Pastores

United States of America

George Patrinos

Netherlands

Andreas Paul

Germany

Chrystal Paulos; Ph.D.

United States of America

Valerio Pazienza

Italy

André Pelegrin

France

Catherine Pellat-Deceunynck

France

Salley Pels

United States of America

David Penetar

United States of America

Wen-Huang Peng

Taiwan

Jeroen Pennings

Netherlands

George Pentheroudakis

Greece

Eduardo Perez-Gomez

Spain

Shira Perl

United States of America

Alessandro Perrella

Italy

Gianluca Perseghin

Italy

Mario Petretta

Italy

Annacarmen Petrizzo

Italy

Brian Petroff

United States of America

Raffaele Pezzilli

Italy

Michael Piagnerelli

Belgium

Vincent Pialoux

France

Gerald Pierard

Belgium

Mario Plebani

Italy

Nikolaus Plesnila

Germany

Claudia Plottel

United States of America

Ryszard Pluta

United States of America

Stergios Polyzos

Greece

Laurentiu M. Popescu

Romania

Luis Porrata

United States of America

Laura Porretti

Italy

Daniel Powell

United States of America

Klaus T. Preissner

Germany

Guilherme Pretto

Brazil

Kari Pulkki

Finland

Silky Punyani

India

Chaim Putterman

United States of America

Chao-Nan Qian

United States of America

Qijun Qian

China

Jie Qian

United States of America

Jianhua Qiu

China

Gh Qiu

Singapore

Jorge Quiroga

Spain

Christoph Rader

United States of America

Laszlo Radvanyi

United States of America

Arash Rafii

Qatar

Gajendra Raghava

India

Osama Rahma

United States of America

Giuliano Ramadori

Germany

Mayur Ramesh

United States of America

Lokinendi Rao

United States of America

Svein Rasmussen

Norway

Ramandeep Rattan

United States of America

Pradip Raychaudhuri

United States of America

Karen Reckamp

United States of America

William Reeves

United States of America

Jörg Reichrath

Germany

Giuseppe Remuzzi

Italy

Jiaqiang Ren

United States of America

Nicholas P. Restifo

United States of America

Sheikh Riazuddin

Pakistan

Giovanni Ricevuti

Italy

Jennifer Richer

United States of America

Daniel Ricklin

United States of America

Ruggero Ridolfi

Italy

Claudia Riedel

United States of America

Gregory Riely

United States of America

Peter Riess

Germany

Gregory Riggins

United States of America

Donata Rimoldi

Switzerland

Neil Riordan

United States of America

Jon Rittenberger

United States of America

Helen Rizos

Australia

Clinton Robbins

United States of America

Bruno Robert

France

David Robertson

Australia

Antonio Rodriguez

Spain

Jose Rojo

Spain

Cristina Romes

United States of America

Rafael Rosell

Spain

Anna Rosell

United Kingdom

Francesca Rossi

Italy

François Rossi

Italy

Claudia Rubie

Germany

Stefan Ruhl

United States of America

Steve Russell

United Kingdom

Mugurel Constantin Rusu

Romania

Marianna Sabatino

United States of America

Sandra Sacre

United Kingdom

Evangelista Sagnelli

Italy

Ryuichi Sakai

Japan

Khashayar Sakhaee

United States of America

Antonio Salas

Spain

Gil Salles

Brazil

Andrea Salmaggi

Italy

Konstantin Salnikow

United States of America

Lobelia Samavati

United States of America

Marta Sanchez-Carbayo

Spain

William Sanders

United States of America

Dario Sangiolo

Italy

Laura Sanz

Spain

Takashi Sasayama

Japan

Seetharama Sastry Konduru

Qatar

Noriyuki Sato

Japan

Alka Saxena

Japan

Dolores J. Schendel

Germany

Erwin Schleicher

Germany

Michael Schmidt

Germany

Ingo Schmidt-Wolf

Germany

Roland Schmitt

Sweden

Roland Schmitz

United States of America

Jonathan Schneck

United States of America

Paul Magnus Schneider

Switzerland

Stephen Schneider

United States of America

Andreas Schober

Austria

Thies Schroeder

United States of America

Hanneke Schuitemaker

Netherlands

Patrick Schuler

Germany

Gary Schwartz

United States of America

Savino Sciascia

Italy

Jean-Yves Scoazec

France

Christina Selinger

Australia

Shantanu Sengupta

India

Mª Jose Serrano

Spain

Gregor Sersa

Slovenia

Somasekar Seshagiri

United States of America

Sana Sfar

Tunisia

Dan Sha

China

James Sham

United States of America

Jingxuan Shan

Qatar

Dejing Shang

China

Anil Shanker

United States of America

Jian-Zhong Shao

China

Orla Sheils

Ireland

Jiangang Shen

Hong Kong

You-Tang Shen

United States of America

Jixin Shi

China

Hiroshi Shiku

Japan

Ashish Chandra Shrestha

Nepal

Galina Shurin

United States of America

Jason Sicklick

United States of America

Harpreet Singh

Germany

Doug Sipp

Japan

Samuel So

United States of America

Paul Sondel

United States of America

Claudio Spada

Italy

Giulio C. Spagnoli

Switzerland

Daniel Speiser

Switzerland

Pasquale Sperlongano

Italy

Geetha Srikrishna

United States of America

Giuseppe Stabile

Italy

Rumen Stefanov

Bulgaria

Ulrike Stein

Germany

Katarzyna Stolarz-Skrzypek

Poland

Peter Storz

United States of America

David Stroncek

United States of America

Ralf Stumm

Germany

Dan Su

China

Jau-Ling Suen

Taiwan

Seigo Sugiyama

Japan

Claudio Sustmann

Germany

Hiroshi Suzuki

Japan

Peter Svec

Slovakia

Arantxa Tabernero

Spain

Masami Takei

Japan

Yuichi Takiguchi

Japan

Giuseppe Tambussi

Italy

Kazuhiro Tamura

Japan

Xiaohua Tan

China

Bee Tan

United Kingdom

Vivek Tanavde

Singapore

Yuanjia Tang

China

Jun-Ming Tang

China

Ping Tang

United States of America

Xao Tang

United States of America

Kai-Fu Tang

China

Hua Tang

China

Parisa Taravati

United States of America

Evangelos Terpos

Greece

Annalisa Terranegra

Italy

Archana Thakur

United States of America

Kelly Jean Thomas

United States of America

John Thompson

Australia

Per Thor Straten

Denmark

Henrik Thorlacius

Sweden

Ling Tian

China

Ingeborg Tinhofer

Germany

Carlo Tocchetti

Italy

Shinsaku Togo

Japan

Rene Tolba

Germany

Sara Tomei

Qatar

Maria Lina Tornesello

Italy

Rosalba Torrisi

Italy

Paolo Tosco

Italy

Hidenori Toyoda

Japan

Lisbeth Tranebjaerg

Denmark

Christopher Triggle

Qatar

Takeshi Tsubata

Japan

Yutaka Tsutsumi

Japan

Sandra Tuyaerts

Belgium

Hirotsugu Uemura

Japan

Takahiro Uenishi

Japan

Kenneth Ugen

United States of America

Mats Ulfendahl

Sweden

Anja Ulmer

Germany

Gudrun Ulrich-Merzenich

Germany

Nadir Ulu

Turkey

Ahmet Tulga Ulus

Turkey

Walter Urba

United States of America

Isao Usui

Japan

Hassan Vahidnezhad

Iran

Bahareh Vali

Canada

Hugo Van Den Berg

United Kingdom

Sjoerd H. Van Der Burg

Netherlands

Jo Van Ginderachter

Bermuda

Inge Van Houdt

Netherlands

Katherine Van Loon

United States of America

Laurens Van Meeteren

Netherlands

Hein Van Poppel

Belgium

Isabelle Van Seuningen

France

Guido Vanham

Belgium

Maria Giuliana Vannucchi

Italy

Tracy Vargo-Gogola

United States of America

Kimberly Varker

United States of America

David Vaudry

France

Sergia Velho

Portugal

Vijayalakhsmi Venkatesan

India

Pedro L Vera

United States of America

Marion Vermeulen

South Africa

Fabio Vescini

Italy

John Vetto

United States of America

Natassia Vieira

United States of America

Elizabeth Vincan

Australia

Maria Luisa Visciano

Italy

Davide Visigalli

Italy

Santiago Viteri

Spain

Leticia Vittone

Argentina

Simona Vittorini

Italy

K. David Voduc

Canada

Paul Walker

Switzerland

Steffen Walter

Germany

Jasmin Walter

Switzerland

Yinsheng Wan

United States of America

Yaohe Wang

United Kingdom

Yingqun Wang

United States of America

Jun Wang

China

Xiangdong Wang

Sweden

Ming-Rong Wang

China

Wei Wang

China

Weimin Wang

China

Xiangdong Wang

China

Yang Wang

United States of America

Shengdian Wang

China

Mong-Heng Wang

United States of America

Tianyi Wang

United States of America

Chris Ward

United Kingdom

Masatoshi Watanabe

Japan

Louise Watson

United Kingdom

Jeffrey Weber

United States of America

Lai Wei

United States of America

Ellen Weinberg

United States of America

Christoph Weiser

Austria

Athol Wells

United Kingdom

Frits Wijburg

Netherlands

Marta Wilhelm

Hungary

Christopher Wingard

United States of America

David Wong

United States of America

Andrea Worschech

Qatar

Yaguang Xi

United States of America

Yunfei Xia

China

Zuolin Xiang

China

Ting Xie

United States of America

Lu Xie

China

Li Xingyi

China

Bin Xiong

China

Ruian XU

China

Zhenzhong Xu

United States of America

Naoki Yamamoto

Japan

Hiroki Yamaue

Japan

Jun Yan

United States of America

Bin Yang

United Kingdom

An-Gang Yang

China

Pan-Chyr Yang

Taiwan

Peizeng Yang

China

Xiao-Feng Yang

United States of America

Victoria Yee

United States of America

Te-Huei Yeh

Taiwan

Sai Yendamuri

United States of America

Jianming Ying

China

Liu Yingbin

China

Keiko Yoshimoto

Japan

Osamu Yoshino

Japan

Yongping You

China

Inchan Youn

Korea, South

Muhammad Zubair Yousaf

Pakistan

Nasser Yousif

United States of America

Hans Yssel

France

Rongjie Yu

China

Jianda Yuan

United States of America

Kevin Yuen

United States of America

Takashi Yugawa

Japan

Anna Zaczek

Poland

Paul Zajac

Switzerland

Gianpaolo Zerbini

Italy

Ben Zeskind

United States of America

Henrik Zetterberg

Sweden

Zhongheng Zhang

China

Liping Zhang

United States of America

Hongquan Zhang

Canada

Xu Dong Zhang

Australia

Yiqiang Zhang

United States of America

Qunzhou Zhang

United States of America

Xulong Zhang

China

Yan Zhang

China

Ziyan Zhao

China

Hong Zhao

China

Jie Zheng

China

Regina Ziegler

United States of America

Anna Linda Zignego

Italy

Lynn Zijenah

Zimbabwe

Niklas Zojer

Austria

Steven Zumbrun

United States of America

